# Erythropoietin supplementation induces dysbiosis of the gut microbiota and impacts mucosal immunity in a non-diseased mouse model

**DOI:** 10.3389/fimmu.2024.1465410

**Published:** 2025-01-23

**Authors:** Guillaume Sarrabayrouse, Corentin Joulain, Stéphanie Bessoles, Andrada S. Chiron, Amine M. Abina, Salima Hacein-Bey-Abina

**Affiliations:** ^1^ Unité des technologies Chimiques et Biologiques pour la Santé, Université Paris Cité, Centre National de la Recherche Scientifique (CNRS), Institut National de la Santé et de la Recherche Médicale (INSERM), UTCBS, Paris, France; ^2^ Clinical Immunology Laboratory, Groupe Hospitalier Universitaire Paris-Saclay, Hôpital Bicêtre, Assistance Publique-Hôpitaux de Paris, Le-Kremlin-Bicêtre, France

**Keywords:** EPO - erythropoietin, mucosal immunity, microbiota, mice model experiments, dysbiosis

## Abstract

A number of drug treatments are known to alter the dialogue between the gut microbiota and the immune system components in the digestive mucosa. Alterations in intestinal homeostasis are now well known to affect peripheral immune responses and favor the occurrence of a number of pathologies such as allergies and cancers. Erythropoietin’s known pleiotropic effects might explain the adverse events sometimes observed in anemic patients treated by erythropoiesis-stimulating agents (ESA). However, the impact of this therapeutic cytokine on the homeostasis of the intestinal tract has not previously been investigated in detail. By studying a mouse model of erythropoietin (EPO) supplementation for 28 days, we observed EPO-induced dysbiosis of the fecal microbiota characterized by a greater bacterial load, lower bacterial diversity and taxonomic changes. With regard to the mucosal immune system, an analysis of leukocyte populations in the small intestine and colon treatment revealed low proportions of ileal CD4 lymphocyte subpopulations (Treg, Tr17 and Th17 cells), IgA-secreting plasma cells, and a major macrophage subpopulation, involved in the control of lymphocyte responses. Our results provide for the first time a descriptive analysis of intestinal EPO’s regulatory properties and raise questions about the involvement of EPO-induced alterations in the microbiota and the gut immune effectors in the control of intestinal and peripheral immune responses.

## Graphical Abstract

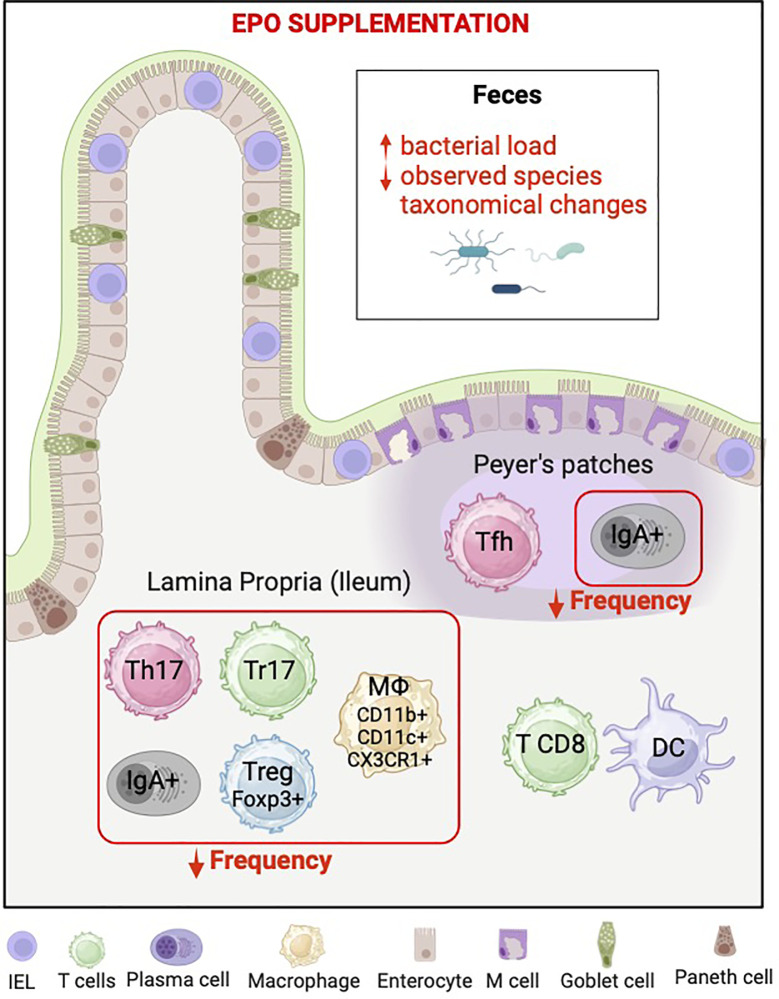

## 1 Introduction

The gut is now known to be a key immunologically active site, and the digestive mucosa has developed unique immune mechanisms that are not found anywhere else in the body. The intestinal barrier consists mainly of a monolayer of epithelial cells, which physically protects intestinal tissues from harmful external factors and provides a microenvironment for the colonization of commensal bacteria while guaranteeing immune tolerance (1). Various immune cells are known to contribute significantly to intestinal barrier function by either interacting directly with epithelial cells or producing immune mediators. Functional mucosal homeostasis of the gut barrier requires not only the intrinsic regulation of intestinal epithelial cells (IECs) but also constant communication with immune cells and gut microbes (2).

Foreign compounds that enter the lumen are taken up by gastrointestinal-associated lymphoid tissues (GALTs), such as Peyer’s patches. Depending on the nature of the antigen, various phenotypically distinct populations of antigen-presenting cells (APCs) are then involved in the orientation of immune responses and the priming of various subpopulations of T lymphocytes (regulatory T cells (Tregs), Th1 cells, Th17 cells, etc.) and B lymphocytes to promote tolerance or inflammation. Furthermore, the intestinal lamina propria (LP) is home to a large number of immunoglobulin A (IgA)-secreting plasma cells. IgA is the most abundant Ig in the gut; it influences the composition of commensal species that colonize the intestinal tract and helps to eliminate pathogens (3).

Erythropoietin (EPO) is a hormone produced by the kidney in response to hypoxia and whose role in erythropoiesis has been well described for many years. EPO has also been characterized as a pleiotropic cytokine that regulates physiological processes in various organs and tissues (4–8). These pleiotropic effects have been attributed to EPO’s ability to bind to and activate different receptors distributed throughout the body and on the surface of various cell types (9–15). EPO’s immunosuppressive role has been widely demonstrated in murine models of supplementation with recombinant human EPO (EPO). Indeed, EPO has been shown to target both innate and adaptive immune cells, induce immune tolerance, and promote the resolution of preclinical autoimmune diseases (16–19). EPO and other erythropoiesis-stimulating agents (ESAs) have been widely used for three decades to correct severe anemia in patients with chronic kidney disease (CKD) or cancer (20). However, from an epidemiological standpoint, it has been reported that EPO/ESAs administration is associated with an elevation of cardiovascular and venous thromboembolism risks in patients (21) and the induction of tumor progression - independently of the type of cancer or the cancer treatment (22, 23). Recently, we demonstrated that EPO affects tumor progression by a direct immunosuppressive action on the tumor microenvironment (24). However, regarding the well-described role of the digestive microbiota on anti-tumor immunity, an indirect effect of EPO on tumor progression through perturbation of the intestinal ecosystem should also be considered.

Previous studies of the impact of EPO on the digestive tract in several rodent models have evidenced a protective effect on the integrity of the intestinal barrier (25, 26). In other studies, EPO has been shown to reduce inflammatory responses and mucosal alterations in models of colitis (27–29), regenerate the colonic mucosa, and enhance antibacterial immune responses in the digestive tract (16, 30). Lastly, only a few studies have assessed the impact of EPO on commensal intestinal bacteria; most of these experimental approaches involved animal models of disease and therefore provided only partial answers (25).

Given the unique immunological characteristics of the gut and the close relationship between the microbiota and gut immunity, we decided to conduct a descriptive pilot study of EPO’s impact on intestinal immunity and the gut microbiota in a non-diseased mouse model of EPO supplementation (31).

## 2 Methods

### 2.1 The experimental animal’ model

1a-Ethics of Animal Use

All animal experiments were performed in accordance with the guidelines of Directive 2010/63/EU and approved by the Ethics Committee for Experimentation of Université Paris Cité and authorized by the French Ministry of National Education, Higher Education and Research (#43255-2023032010258854). Female C57BL/6 mice (Envigo RMS SARL, Harlan Laboratories, Gannat, France) were housed in cages (5 per cage) and kept at 22 ± 2 °C with a 12:12 h light/dark cycle, in accordance with European Union guidelines.

1b-Mice treatment and management

**Figure 1:**
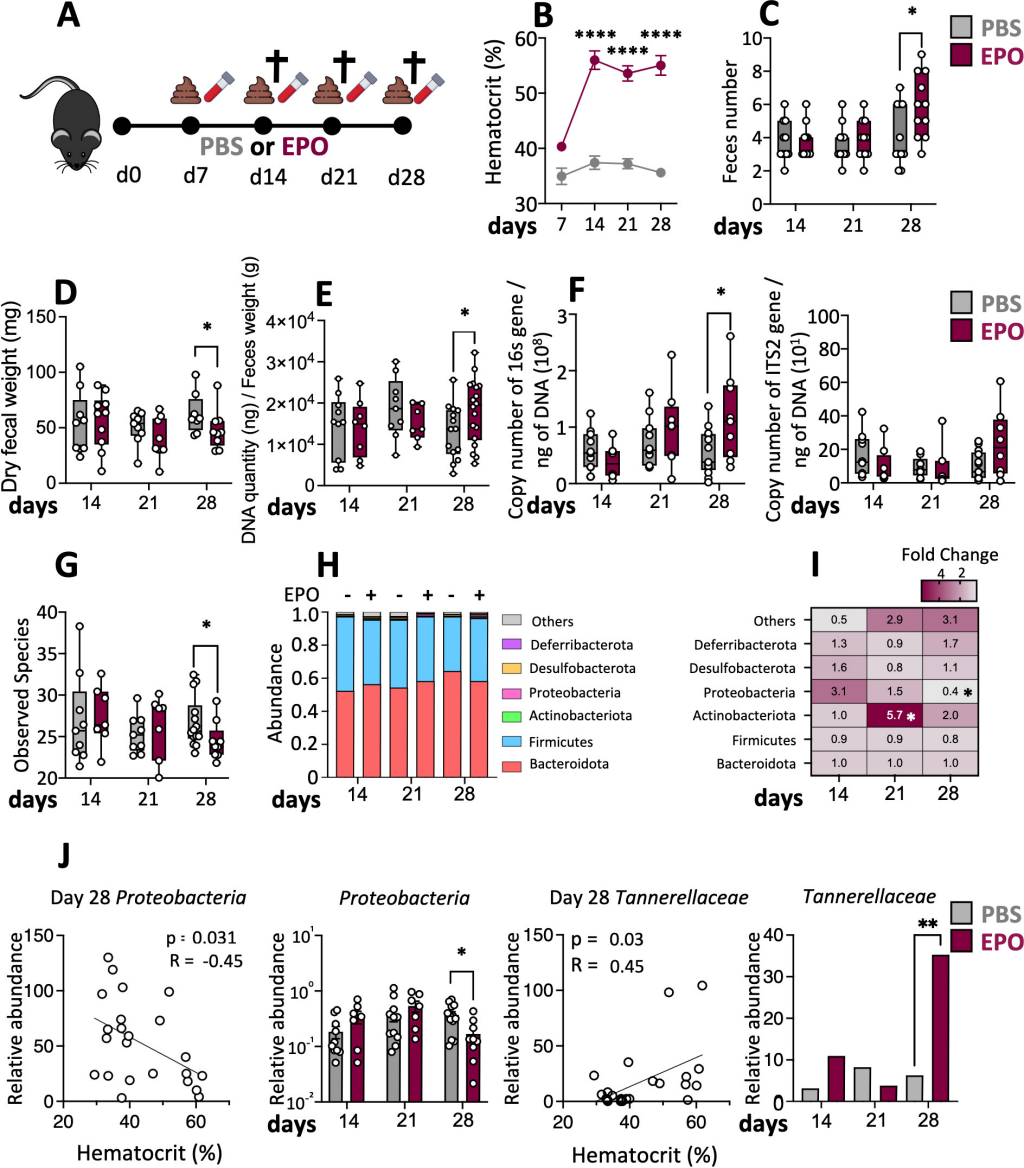
Changes in the fecal microbiota induced by EPO treatment. **(A)** C57Bl6 female mice were treated with 40 IU of EPO or with PBS for 4 weeks. Blood and stool samples were collected on days 7, 14, 21 and 28 after the start of treatment. Mice were euthanized on days 14, 21 or 28 and tissue samples were collected for analysis. **(B)** The hematocrit on days 7, 14, 21 and 28 after the start of treatment (*n* > 15) (Mann–Whitney test). **(C)** Quantity of feces produced by EPO-treated mice and control animals (*n* > 10) (Mann–Whitney test). **(D)** Fecal samples were collected, desiccated and weighed (*n* > 10) (Mann–Whitney test). Genomic DNA from mouse feces was extracted and **(E)** microbial density evaluated (*n* > 15) as well as **(F)** 16S rRNA copy number (bacterial load) or the ITS2 copy number (fungal load) (*n* > 10) (Mann–Whitney test). **(G)** Box plots of the Observed species index of the microbiota in the mouse feces (*n* > 10). **(H)** The microbial composition at the phylum level. **(I)** Heatmap of fold differences in phylum abundance between EPO-treated and control mice (*n* > 10). **(J)** Spearman correlations between bacterial abundance and the hematocrit and relative abundances of the phyla *Proteobacteria* and the family *Tannerellaceae* in EPO-treated mice and control mice (Kruskal-Wallis). False discovery rate (FDR)-corrected *P*-values. FDR < 0.05 was considered significant for all tests *p < 0.05; **p < 0.01; ****p < 0.0001. For each parameter studied, on average four independent experiments were carried out, each involving 3 to 5 mice per group.

In each experiment, groups of ten 6-week-old female mice received a subcutaneous injection of either 40 units of EPO (Eprex 10,000 IU/mL, Janssen) or PBS vehicle three times a week for 4 weeks. Caudal vein blood was collected in an EDTA tube once a week during the treatment period, and the complete blood count (CBC) was determined on an automatic hematology analyzer (MS9, Melet Schloesing Laboratories) (**Figure 1A**). An increase in hematocrit (Hct) was used as a marker to assess the biological effectiveness of EPO supplementation and as a criterion for including mice in the study. This biological validation ensures that the effects observed in the gut (compared to control mice) occur in a context of confirmed erythropoietin bioavailability, with its action on erythropoiesis serving as evidence of both the efficacy of the injections and the physiological responsiveness of the mice (**Figure 1B**; **Supplementary Table 1**). Mice from both groups were euthanized 14, 21 and 28 days after the start of the course of treatment. At the time of euthanasia, stools were collected, and spleens and gut (Peyer’s patches, ileum, and colon) were isolated, weighed and studied to assess changes over time in gut mucosal immunity (tissue samples) and the composition of the fecal microbiota (stool samples) (**Figure 1A**). It should be noted that the data generated are from several groups of animals followed and treated over 28 days then euthanized at different times as previously indicated. Given the technical/analytical diversity of the studies carried out, the requirement for handling over a short time, and the technical platform access constraints, it was preferable to conduct experiments on reasonably sized groups of mice (3–5 mice per group for each condition), ensuring that each EPO-treated group was analyzed in comparison to a control group (PBS) euthanized on the same day.

### 2.2 Fecal sample water content

Samples were collected in pre-weighed collection tubes and weighed to determine their mass. To measure the water content of a sample, open tubes were heated at 65°C for 24 h and reweighed (32). The water content of a sample was calculated as the difference between the initial and final masses, divided by the initial mass.

### 2.3 Fecal sample collection and genomic DNA extraction

Immediately after collection, fecal samples were frozen and stored at −80°C. Genomic DNA was extracted in accordance with the International Human Microbiome Standards (IHMS), using the standardized operational procedure number 7 (SOP7) (https://human-microbiome.org). Briefly, a frozen aliquot of each sample was thawed and suspended in 250 μl of guanidine thiocyanate, 40 μl of 10% N-lauroyl sarcosine, and 500 μl of 5% N-lauroyl sarcosine. Genomic DNA was extracted by mechanical disruption of the microbial cells with glass beads (0.1 mm, Sigma-Aldrich) using a TissueLyser III (QIAGEN), and nucleic acids were recovered from clear lysates by alcohol precipitation as previously described (33).

The concentration and purity of each gDNA sample obtained are assessed using a nanodrop (Nanodrop One, Thermo Fisher Scientific) and the quality of the DNA is assessed by means of a genomic gel (1% agarose, 100 volts, 1 hour) as recommended by the IHMS. Any sample showing poor purity or excessive degradation is excluded from the study.

### 2.4 Microbial load analysis

Extracted genomic DNA was used to amplify the V4 region of the 16S rRNA gene or the ITS2 region by quantitative real-time PCR, using universal primers for counting the microbial load and specific primers for targeting fungi, as described by Sarrabayrouse et al. (34).

Blank controls were used to assess the absence of sample contamination, a positive control for repeatability was used and PCR efficiency was evaluated by calculating the standard curve using the CFX maestro 3.0 software (BioRad).

### 2.5 Composition of the microbiota

The V4 region of the 16S rRNA gene was amplified for 30 cycles (using an annealing temperature of 65°C, Taq polymerase MTP (Sigma Aldrich), and the primers V4F_517_17 (5′‐GCC AGC AGC CGC GGT AA‐3′) and V4R_ 805_19 (5′‐GAC TAC CAG GGT ATC TAA T‐3′) (35). PCR amplicons were purified and their quality and concentration were checked before sequencing on an Illumina MiSeq platform (Genotoul, Toulouse). The raw 16S rRNA sequences were analyzed using the Find Rapidly OTU with Galaxy Solution bioinformatics pipeline (FROGS) (INRAE, France) as well as the web application easy 16S (INRAE, France) to visualize and analyze amplicon sequencing metagenomics data as described previously (36).

### 2.6 Preparation of intraepithelial lymphocytes and Lamina Propria mononuclear cells

Mice were euthanized by cervical dislocation and the entire intestinal tract was removed. The intestinal and colonic contents were discarded. The ileum and colon were excised and opened longitudinally. Peyer’s patches were collected, and tissue was weighed and then washed with calcium- and magnesium- free Hank’s balanced salt solution (HBSS) (11560616; Fisher scientific). The washed ileum and colon were cut into small pieces and incubated with HBSS containing 1 mM DL-Dithiothreitol (43816; Sigma-Aldrich) and 5 mM EDTA (E8008; Sigma-Aldrich) for 8 min at 37°C. To remove the epithelial layer, tissues were washed three times in HBSS containing 5 mM of EDTA and epithelial compartments were collected by filtration through a 100-micron cell strainer. The pieces of mucosa were then washed in HBSS and dissolved by incubation in HBSS containing 1.5% fetal bovine serum (CVFSVF06-01; Eurobio Scientific), 1.0 mg/mL collagenase D (11088858001; Merck), and 0.1 mg/mL DNase (10104159001; Merck) for 30 min at 37°C. The solution was centrifuged, filtered through a 40-micron cell strainer, the resulting pellet was resuspended, and the cells were counted and analyzed.

### 2.7 Flow cytometry

In a cell viability assay, two million cells were stained for 30 min with Fixable Viability dye eFluor 506 (65-0866-18, eBioscience). After blocking with anti-FcR (553141, BD Biosciences) for 5 min, the cells were incubated for 30 min with a specific fluorescence-labeled antibody diluted in fluorescence-activated cell sorting (FACS) buffer solution (PBS-FCS 5%, EDTA 5 nM). The surface antigens of isolated cell suspensions were stained with the antibodies described in the **Supplementary Material**. For intracellular staining, cells were permeabilized using a fixation/permeabilization solution (00-5523-00; eBioscience) before intracellular staining with antibodies (described in the **Supplementary Material**). Flow cytometry antibodies were purchased from BioLegend, ebioscience and Miltenyi. The Fortessa FACS system (BD Biosciences) was used. The flow cytometry results were analyzed using FlowJo™ v10.8 Software (BD Life Sciences). The gating strategies for FACS analysis and detailed information on the antibodies used in this study are respectively summarized in **Supplementary Figure 1** and the **Supplementary Material**.

### 2.8 Evaluation of cytokine expression by activated Lamina Propria Lymphocytes

For functional assays, 1.5 millions/mL LPL were placed in RPMI (Gibco) + 10% FCS (Eurobio), penicillin/streptomycin (Gibco), 50ng/mL PMA (Merck) and 1 microg/mL ionomycin (Merck) for 4h. After the first hour of activation, brefeldin (Fisher Scientific) was added. At the end of the activation protocol, cells were washed in PBS (Fisher Scientific) for staining. One million cells were stained for 30 min with Fixable Viability dye eFluor 506 (65-0866-18, eBioscience). After blocking with anti-FcR (553141, BD Biosciences) for 5 min, the surface antigens of stimulated cells were stained with the antibodies described in the **Supplementary Materials**. For intracellular cytokine staining, cells were permeabilized using a fixation/permeabilization solution (00-5523-00; eBioscience) before staining with anti-IL10 and/or anti-IL17A antibodies (described in the **Supplementary Material**). Gating strategies for FACS analysis are summarized in **Supplementary Figure 2**.

### 2.9 Statistics

Statistical analyses were carried out in FROGS, easy 16s (R Foundation for Statistical Computing), and GraphPad Prism version 10 (GraphPad Software). Shapiro-Wilk and Anderson-Darling tests were used to check normality of data distribution. Data were not normally distributed, so nonparametric tests were used. We compared quantitative and categorical variables between 2 unpaired groups with the Mann-Whitney *U* test (*p < 0.05; **p < 0.01; ***p < 0.001, ****p < 0.0001). The Kruskal–Wallis one-way test of variance was used to compare the median number of sequences of the groups at various taxonomic levels. When possible, the analysis provided false discovery rate (FDR)-corrected *P*-values. FDR < 0.10 was considered significant for all tests (Benjamini-Hochberg method). To determine the association between quantitative data, we performed Spearman’s rank correlation coefficient.

## 3 Results

### 3.1 EPO supplementation impacts the fecal microbiota

Over the course of treatment, there were no significant differences between EPO-treated animals and control animals in terms of macroscopic variables like bodyweight, cecum weight, and ileum and colon lengths (**Supplementary Figure 3**). On day 28, the EPO-treated group had a significantly higher mean stool frequency and a significantly lower mean stool weight, when compared with the control group (**Figures 1C, D**). Given that stool frequency and consistency have been linked to the composition of the gut microbiota (34), we compared the microbiota in EPO-treated mice vs. control-treated mice. First, we observed that the fecal microbial density increased following 28 days of EPO treatment (**Figure 1E**). We next explored the fecal bacterial and fungal loads using PCR amplification of the 16S rRNA gene and the ITS2 region respectively. The bacterial load in stools was significantly higher in EPO-treated mice than in control mice (**Figure 1F**) but there was no intergroup difference in the fungal load (**Figure 1F**). A 16S sequence analysis revealed a low level of bacterial diversity (observed species) in feces on day 28 of EPO supplementation mice (**Figure 1G**). Other diversity indexes such as Chao1 or Shannon, as well as analysis of beta diversity, failed to identify any significant differences between the treated and the control groups (data not shown). We next looked at changes of time in fecal microbiota composition at the phylum level. The EPO-treated and control groups did not differ significantly in the relative abundance of the two major phyla *Bacteroidota* and *Firmicutes* (**Figures 1H, I**; **Supplementary Figure 4**) or the minor phyla *Desulfobacterota* and *Deferribacterota*, (**Figure 1I**). In contrast, the relative abundance of the *Actinobacteriota* was significantly higher in the EPO-treated group on day 21 and (albeit to a lesser extent) on day 28 (**Figure 1I**; **Supplementary Figure 4**). The abundance of the *Proteobacteria* started to decrease on day 21 in the EPO-treated group, and the difference vs. the controls was significant on day 28 (**Figures 1I, J**). Intergroup comparisons of the fecal microbiota composition at other taxonomic levels revealed a particular feature: a significantly higher abundance of *Tannerellaceae* at the family level in the EPO-treated group on day 28 (**Figure 1J**). In order to take account of interindividual differences in the response to EPO treatment, we used the hematocrit as a marker of effectiveness. Spearman’s test revealed a number of correlations between several microbial phyla and families in the fecal samples and the hematocrit over the EPO treatment period (**Figure 1J**; **Supplementary Figure 5**). The hematocrit was positively correlated with the relative abundances of *Clostridia UCG-014* and *Rikenellaceae* on day 21 and *Muribaculaceae*, *Oscillospirales* and *Tannerellaceae* on day 28. In contrast, the Hct was negatively correlated to relative abundances of *Proteobacteriota*, *Clostridia vadinBB60* and *Erysipelotrichaceae* on day 28. (**Figure 1J**; **Supplementary Figure 5**). Taken as a whole, our data suggest that EPO-induced dysbiosis developed progressively in EPO-treated animals.

### 3.2 EPO impacts mucosal immunity

**Figure 2:**
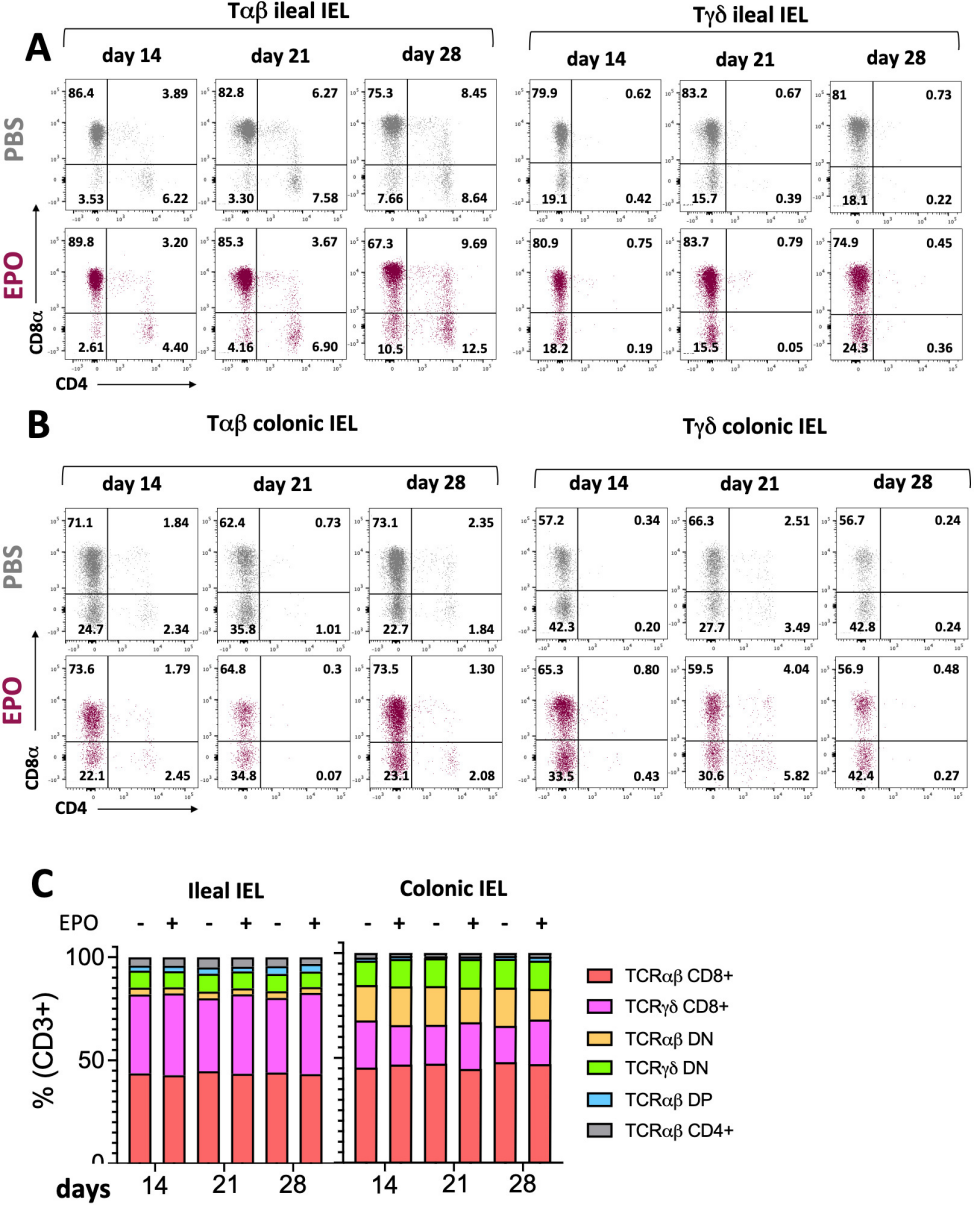
Impact of EPO on Intra Epithelial T cells subpopulations. **(A)** Representative dot plots of Tαβ and Tγδ ileal IELs in EPO-treated mice and control (PBS-treated) mice, after 14, 21 and 28 days of treatment. **(B)** Representative dot plots of Tαβ and Tγδ colonic IELs in EPO-treated mice and control (PBS-treated) mice, after 14, 21 and 28 days of treatment. **(C)** The proportions of the main IELs populations among CD3-positive cells in PBS- and EPO-treated mice, after 14, 21 and 28 days of treatment (*n* > 10). For each parameter studied, on average four independent experiments were carried out, each involving 3 to 5 mice per group.

**Figure 3:**
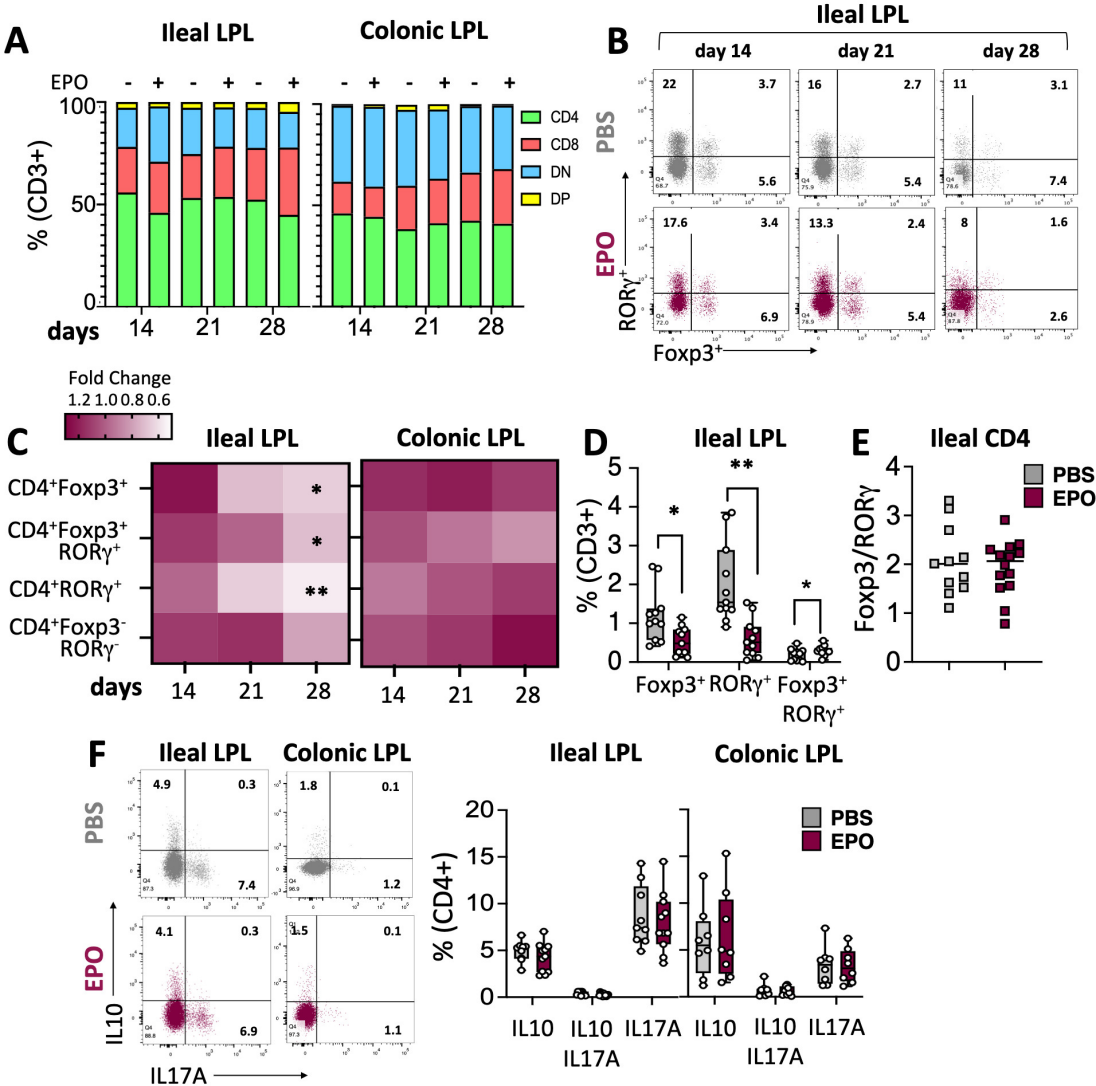
Impact of EPO on Lamina Propria T cells subpopulations. **(A)** The proportions of the main LPLs populations among CD3-positive cells in PBS- and EPO-treated mice, after 14, 21 and 28 days of treatment. **(B)** Representative dot plots of Foxp3 and RORγ transcription factor expression by ileal CD4^+^ cells in EPO-treated mice and control (PBS-treated) mice, after 14, 21 and 28 days of treatment. **(C)** Heat map of the fold differences between EPO-treated mice and control mice in the proportions of CD4^+^Foxp3^+^, CD4^+^RORγ^+^, CD4^+^Foxp3^+^RORγ^+^, CD4^+^Foxp3^-^RORγ^-^ cells among CD3^+^ cells from the ileal and colonic LP, after 14, 21 and 28 days of treatment. **(D)** Frequency of CD4^+^Foxp3^+^, CD4^+^RORγ^+^, CD4^+^Foxp3^+^RORγ^+^ populations among ileal CD3^+^ cells in mice treated with PBS or EPO, after 28 days of treatment (*n* > 10). **(E)** Foxp3/RORγ ratio in ileal CD4+ T lymphocytes from control (PBS) and EPO-treated groups (*n* > 10). **(F)** Representative dot plots and frequencies of IL10+, IL17A+ and IL10+IL17A+ ileal and colonic CD4^+^ T lymphocytes following 6h of PMA/Ionomycin stimulation (*n* = 10 for ileal samples; *n* = 8 for colonic samples). For each parameter studied, on average four independent experiments were carried out, each involving 3 to 5 mice per group (except for the cytokine expression study including two experiments). Each symbol corresponds to an individual mouse. The data are expressed as the mean ± SD. Mann-Whitney tests. *p < 0.05; **p < 0.01.

**Figure 4:**
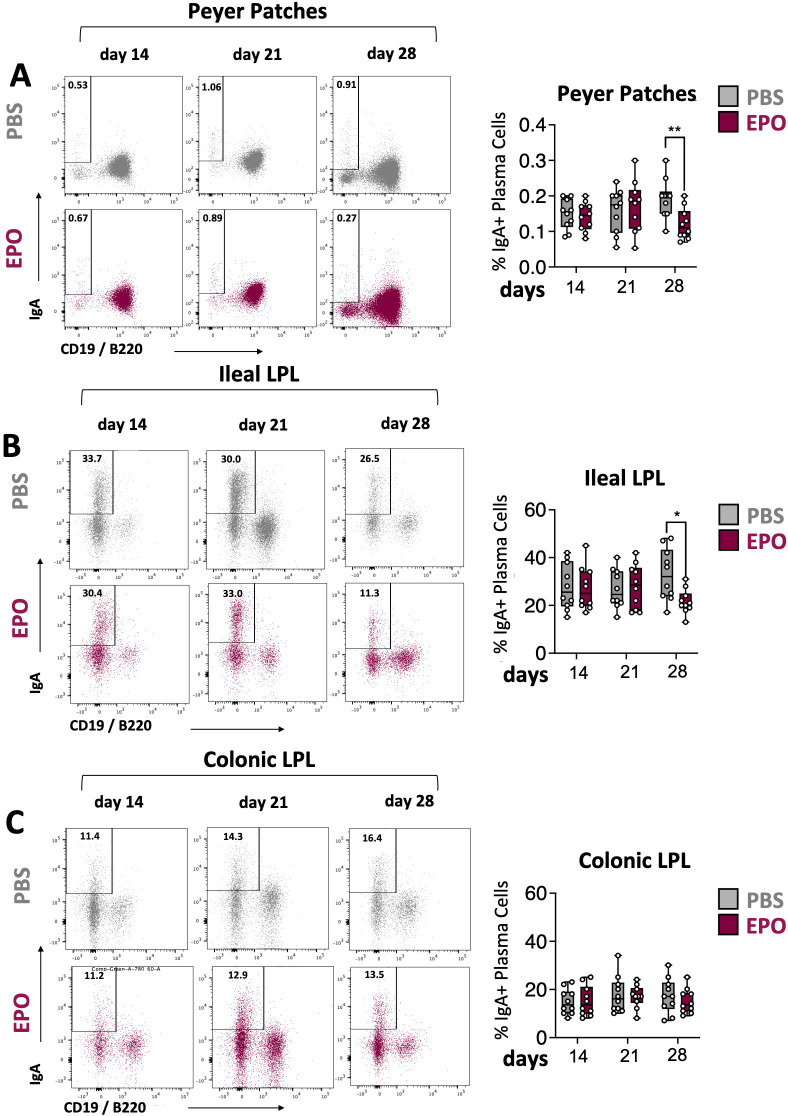
Impact of EPO on mucosal IgA+ plasma cells. Representative dot plots and frequencies of IgA+ plasma cell populations in Peyer’s patches **(A)**, ileal **(B)** and colonic **(C)** LP of mice treated with PBS and EPO, after 14, 21 and 28 days of treatment. IgA+ plasma cell population is derived from the lineage negative CD3-CD11c-NK1.1- parent population (see **Supplementary Figure 1D**). For each parameter studied, on average four independent experiments were carried out, each involving 3 to 5 mice per group. Each symbol corresponds to an individual mouse. The data are expressed as the mean ± SD. Mann-Whitney tests. *p < 0.05; **p < 0.01.

**Figure 5:**
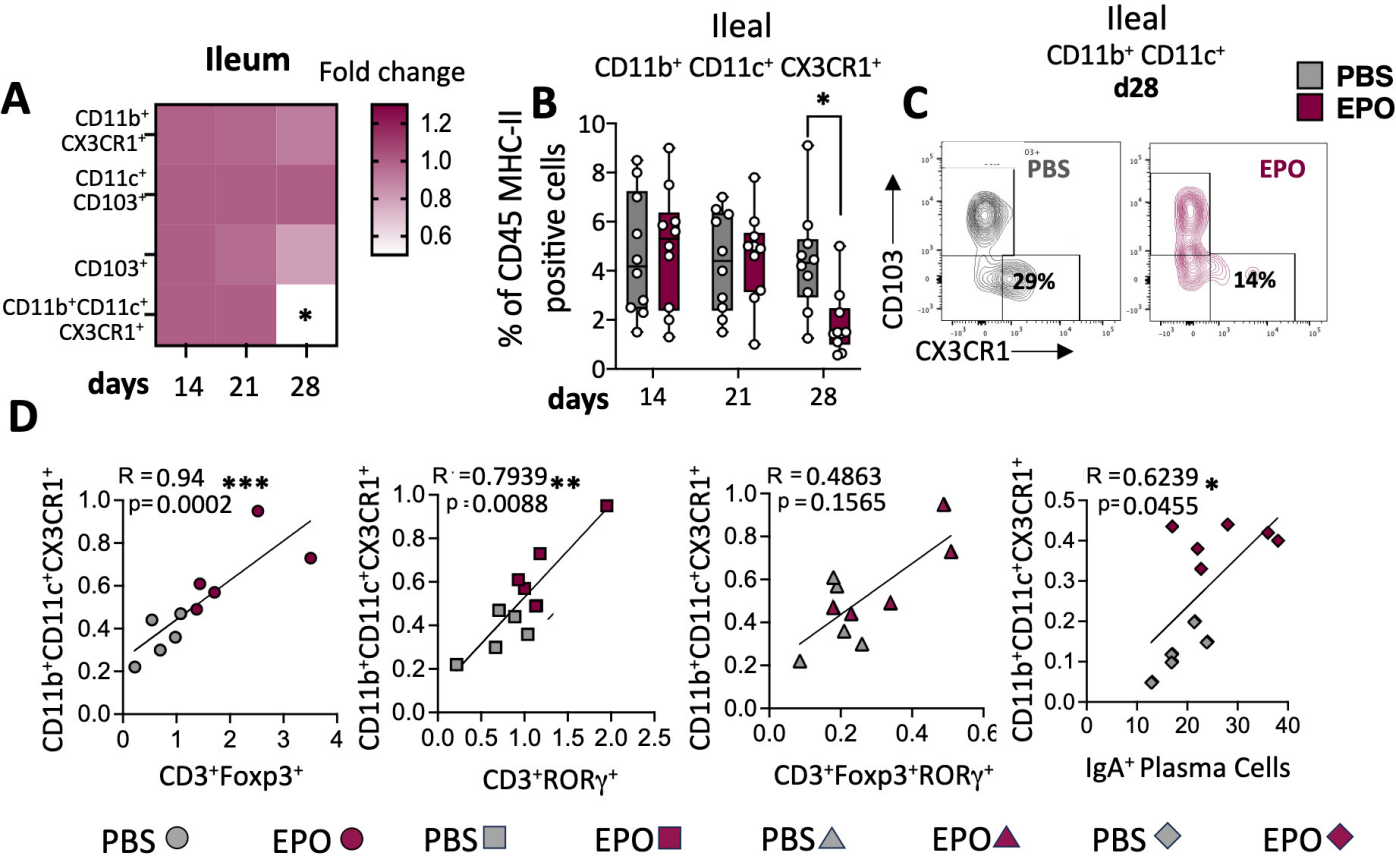
Impact of EPO on mucosal myeloid APC. **(A)** Heat map of the fold differences between EPO-treated mice and control (PBS-treated) mice in the proportions CD11b^+^CX3CR1^+^, CD11c^+^CD103^+^, CD11b^+^CD11c^+^CX3CR1^+^ and CD11b^+^CD11c^+^CD103^+^ among CD45^+^MHC-II^+^ APC cells of the ileal LP, after 14, 21 and 28 days of treatment. **(B)** The frequency of CD11b^+^CD11c^+^CX3CR1^+^ population among ileal LP APCs from PBS- and EPO-treated mice, after 14, 21 and 28 days of treatment. **(C)** Representative dot plots of CD103 and CX3CR1 marker expression among CD11b^+^CD11c^+^ cells in the ileal LP of mice treated for 28 days with PBS or EPO (*n* = 10). **(D)** Spearman’s coefficient for the correlation between CD11b^+^CD11c^+^ CX3CR1^+^ cells and lymphocytes subpopulations in the ileal LP. Each symbol corresponds to an individual mouse (*n* = 10); grey symbols are representative of PBS treated mice and marron symbols are representative of EPO treated mice. For each parameter studied, on average four independent experiments were carried out, each involving 3 to 5 mice per group. The data are expressed as the mean ± SD. Mann-Whitney tests and spearman correlations. *p < 0.05; **p < 0.01; ***p < 0.001.

We next used multiparameter flow cytometry approaches to investigate the impact of EPO treatment on mucosal immune cell subpopulations in the ileum and colon (**Supplementary Figure 1**). Mucosal T lymphocyte subpopulations are located both in the epithelium that lines the digestive mucosa (IELs) and in the LP, which is the connective tissue beneath the epithelium (LP leukocytes (LPLs)). CD3^+^ IELs can be divided into two subpopulations regarding the expression of the alpha-beta (αβ) or gamma delta (γδ) TCR. In agreement with the literature data, we observed intergroup differences in the frequency of ileal and colonic CD3^+^ IEL subpopulations (**Figure 2**; **Supplementary Figure 6**). In control animals, double-negative (DN) CD4^-^CD8^-^ αβ and γδ T cells are more frequent among colonic IELs, whereas CD8^+^ γδ T cells are more frequent among ileal IELs. Whatever the duration of the treatment (14, 21 or 28 days), EPO did not appear to be associated with differences in the frequency of ileal or colonic Tαβ and Tγδ IEL subpopulations (**Figure 2**; **Supplementary Figure 6**). Regarding LPLs, no significant intergroup differences in the distribution of ileal and colonic CD3^+^ subpopulations (CD4^+^, CD8^+^, CD4^-^CD8^-^, CD4^+^CD8^+^) were observed on days 14 and 21 of the EPO treatment (**Figure 3A**; **Supplementary Figure 7A**). In contrast, significantly lower frequencies of CD4^+^Foxp3^+^ (Treg), CD4^+^RORγ^+^ (Th17) and CD4^+^Foxp3^+^RORγ^+^ (Tr17) lymphocyte populations were observed specifically among ileal CD3^+^LPLs on day 28 (**Figures 3B–D**; **Supplementary Figures 7B, C**) but without affecting the Treg/Th17 ratio (Foxp3/RORγ ratio), as shown in **Figure 3E**. The cells’ ability to produce cytokines was also assessed, showing no impact of EPO treatment on IL10 and IL17A production in ileal and colonic CD4^+^ LPLs (**Figure 3F**). IgA-positive plasma cells [described phenotypically as a CD3^-^ CD11c^-^ NK1.1^-^ CD19^-^ B220^-^ population expressing IgA (**Supplementary Figure 1D**)] are the main players in mucosal humoral immunity and therefore control gut homeostasis. They are found in the LP and in ileal Peyer patches; the latter form a secondary lymphoid organ within which, (i) gut ileal B cells achieve IgA class switching and, (ii) plasma cells mature with the help of follicular helper T lymphocytes (Tfh). On day 28 of treatment, we observed significantly lower frequencies of IgA+ plasma cells in Peyer’s patches and in the ileal LP of the EPO-treated group, relative to controls (**Figures 4A, B**). In contrast, EPO treatment did not appear to influence the proportion of plasma cells from the colon (**Figure 4C**) and no intergroup differences in the frequencies of Peyer’s patches TfhCD4^+^ (CD3^+^CD4^+^CD44^+^CXCR5^+^PD1^+^) and TfhCD8^+^ (CD3^+^CD4^-^CD44^+^CXCR5^+^PD1^+^) cells were observed (**Supplementary Figure 8**). The influence of EPO treatment on these key effectors of the adaptive immune response prompted us to evaluate the hormone’s action on antigen-presenting cells from the ileal and colonic LP. As shown in **Supplementary Figure 9A**, EPO treatment was not associated with a difference in the total frequency of APCs (CD3^-^CD19^-^NK1.1^-^CD45^+^MHC-II^+^) in the ileum LP or the colon LP. Dendritic cells (DCs) and macrophages (the mains APCs in the intestinal tract) bear a specific set of markers. Lamina propria DCs have a CD11c^+^CD11b^-^CX3CR1^-^CD103^+^or CD11c^+^CD11b^+^CX3CR1^-^CD103^+^ phenotype, whereas mucosal macrophages are more related to CD11b^+^CD11c^-^CX3CR1^+^CD103^-^ or CD11b^+^CD11c^+^CX3CR1^+^CD103^-^ cells (**Supplementary Figure 1C**). As shown in **Supplementary Figures 9B, C**, the frequency of DC and macrophage subpopulations in control animals was quite similar in the ileal and colonic mucosa. On day 28 of EPO treatment, we observed significantly lower frequency ileal macrophages expressing the CD11b^+^CD11c^+^CX3CR1^+^CD103^-^ phenotype (**Figures 5A–C**). There were no intergroup differences in the other APC subpopulations, whatever the time point (**Supplementary Figure 9**). Lastly, we observed a positive correlation between the frequency of CD4^+^Foxp3^+^, CD4^+^RORγ^+^ T lymphocytes, IgA^+^ plasma cells and the ileal CD11b^+^CD11c^+^CXC3CR1^+^ macrophage population in the EPO-treated group only (Spearman’s correlation test, **Figure 5D**). It is noteworthy that this macrophage population is reportedly involved in the differentiation of Th17, Tregs and plasma cells (37–42). Overall, our phenotypic analysis of gut mucosal immune cells evidenced significant differences in the frequencies of adaptive and innate ileal effectors in EPO-supplemented mice, relative to controls.

## 4 Discussion

The results of our pilot study evidenced an impact of EPO supplementation on digestive homeostasis. Although EPO is known to have an immunosuppressive role and has been widely studied in various pathological contexts, the hormone’s specific action on the unique features of gut immune effectors has not been well characterized. The only studies concerning the effect of EPO on the gut highlighted changes in intestinal barrier integrity (25–27), pathogen clearance (16, 30), inflammation (28, 29) and the composition of the commensal microbiota (25). However, most of these results were obtained in disease models and involved particular EPO doses and administration routes. Our study was designed to evaluate EPO’s impact on the digestive tract in a healthy mouse model and therefore determine whether EPO has previously unknown functions in the unique gut immune environment, involving symbiosis with the commensal microbiota.

With regard to physiology, our study revealed EPO-associated differences in fecal water content, and intestinal transit. Indeed, some rhEPO-treated patients experience repeated episodes of diarrhea (43). The difference in water content might be associated with EPO’s role in the reabsorption of water across the colonic mucosa. This reabsorption is dependent on ATP pump activity and is known to be downregulated by activation of the JAK2 kinase (44) involved in the EPO-receptor-initiated signaling cascade. Gut motility and transit are partly dependent on the digestive tract’s smooth muscle, the myocytes of which are known to express the EPO receptor (45). The accelerated intestinal transit observed in mice might therefore be a direct consequence of EPO’s putative effect on intestinal smooth muscle cells; however, this supposition has not been proven.

Changes in fecal consistency and intestinal transit are known to be associated with alterations in the richness and composition of the fecal microbiota (46). Our results demonstrated that the dysbiosis associated with 28 days of EPO treatment is characterized by (i) a greater bacterial load, (ii) lower bacterial diversity, and (iii) taxonomic changes with a significantly higher abundance of *Actinobacteriota* and *Tannerellaceae* and a lower abundance of *Proteobacteria*. Changes in fecal bacterial load and diversity have already been reported in many disease settings and appear to be linked to changes in digestive tract immunity (47). Low bacterial diversity is a common feature of inflammatory bowel disease (48, 49). On the taxonomic level, several studies have identified *Proteobacteria* as a possible microbial signature of disease (50–52). An elevated abundance of *Actinobacteriota* has been identified in patients with inflammatory bowel disease (53), whereas an elevated abundance of *Tannerellaceae* has been found in preclinical models of colonic adenocarcinoma (54), and allergy (55). These results show that EPO treatment induces dysbiosis of the gut microbiota, which displays taxonomic similarities with several disease states. It must be borne in mind that interplays between host genetic factors and dysbiosis has an essential role in the pathogenesis of several diseases (56).

With regard to gut mucosal immunity, our results highlighted changes in the frequency of intestinal resident immune cells after 28 days of EPO supplementation. Our observations are not fully in line with the literature data on the immunomodulatory impact of EPO, although this disparity might be due (at least in part) to the specific features of mucosal immunity in the gut. Firstly, IELs collaborate with epithelial cells to shape the intestinal barrier. In the healthy state, the gut LP contains many types of myeloid and lymphoid cells that maintain tolerance or implement inflammatory responses.

There were no differences between EPO-treated mice and control mice in the frequencies of the main mucosal T lymphocyte subpopulations (IELs) in the ileum and the colon. These cells constitute the first line of defense against intestinal pathogens (57). However, we analyzed broad subpopulations of IELs, and a more detailed phenotypic study might be useful for identifying potentially EPO-dependent changes in these complex cell networks.

In contrast to the findings for IELs, LPL frequencies were impacted by EPO treatment. Significantly lower frequencies of CD4^+^ T cell subpopulations (Foxp3^+^ Tregs, Th17 cells, and Tr17 cells) and IgA^+^ plasma cells were observed in the ileal LP of EPO-treated mice. The induction and maintenance of the various lymphocyte populations depends on different subsets of APC. In the healthy state, intestinal DCs are considered to be tolerogenic and participate in the induction of Foxp3^+^ regulatory T cells (iTregs). However, other subtypes of intestinal DCs can induce Th1 (CD103^+^CD11b^-^) (58), Th2 (CD103^+^CD11b^+^) (59) or Th17 (CD103^-^CD11b^+^) (60) populations. Our study did not reveal an association between EPO treatment and intestinal DC subpopulations. In contrast, EPO treatment was associated with a low frequency of a macrophage subpopulation (CX3CR1^+^, MHC-II^+^, CD11b^+^, CD11c^+^) in the ileal LP (61).

In functional terms, this macrophage subpopulation can reportedly eliminate microbes and apoptotic cells, remodel tissues, produce IL-10 (62), and ensure the continued local stability of mucosal Treg populations. Furthermore, these macrophages are able to capture bacteria from the digestive tract and then transfer their antigens to DCs, which in turn induce Tregs and Th17 lymphocytes (38). Lastly, other studies suggest that CXC3CR1^hi^ macrophages are involved in the regulation of IgA^+^ plasma cell differentiation (41). Given the diverse functions performed by this macrophage population, the low frequency observed in EPO-supplemented mice suggests the presence of various cascading effects on ileal LPL induction or differentiation. Hence, the significantly low frequencies of CD4^+^ Foxp3^+^ Tregs, CD4^+^ RORγ^+^ Th17 lymphocytes, and IgA^+^ plasma cells in the ileal LP in EPO-supplemented animals might be a consequence (at least in part) of the EPO-induced relative decrease in CX3CR1^+^ macrophage frequency. This hypothesis is supported by the observed correlations between the number of these macrophages and each of the other three cell populations in the ileal gut.

While the low frequency of Th17 cells observed in our EPO-treated mice is in line with the literature data (63), the low frequency of Foxp3^+^ Tregs is not (17). Indeed, extensive work by Cravedi and colleagues has shown that EPO promotes Treg expansions. Our results suggest that the involvement of the ileal CX3CR1^+^ macrophage population counteracts the previously documented direct effect of EPO on the Tregs, resulting in a reduction in the proportion of these cells in the ileum. This part of our results sheds new light on the complexity of EPO’s pleiotropic functions; by influencing the cooperation between several cell populations, EPO can ultimately manifest effects that contradict those produced directly on a given cell population.

One must also consider the specific environment of the intestinal mucosa, which might account for the effects of EPO not previously described in disease settings or for immune responses that do not involve the gut. More generally, our results raise the important question of whether the EPO-associated differences in immune effectors in the ileum are related to changes in the composition of the microbiota. It is conceivable that a change in the microbiota might direct the immune response. Indeed, the intestinal microbiota produces a large number of components capable of conditioning the phenotype of mucosal APCs and thus directing T cell responses (64). Niess et al.’s report of low numbers of intestinal CX3CR1^+^ APCs in germ-free mice highlighted the importance of microbiota in controlling the cells’ frequency (65). Thus, the low bacterial diversity associated with EPO treatment might cause changes in CX3CR1^+^ macrophage frequency and might explain the unexpectedly low frequency of intestinal Foxp3^+^ CD4^+^ Tregs observed in EPO-treated mice. However, our results do not suggest that CX3CR1^+^ cells are involved in the EPO-associated depletion of Tr17 regulatory cells. Although the nature of the APCs involved in the differentiation of these cells is still subject to debate, their induction by the intestinal microbiota is well-documented (66). Indeed, germ-free and antibiotic-treated mice have a low proportion of Tr17 cells, and Sefik et al. demonstrated that bacteria belonging to several phyla, including the *Proteobacteria* (66) were involved in the induction of these cells. Thus, the changes in the gut microbiota observed in our EPO-treated mice might be linked to the low observed frequency of Tr17 cells.

In conclusion, while our results clearly demonstrate the effects of EPO on both the fecal microbiota and intestinal immunity, they do not establish a direct cause-and-effect relationship between these two phenomena, which represents a limitation of this study. To address this, future research involving fecal microbiota transplantation from EPO-treated mice into untreated mice will enable us to directly evaluate the contribution of EPO-induced dysbiosis to the immune alterations observed in the lamina propria. Further analysis of the interplay between intestinal commensal microbiota and the immune system in the digestive tract is essential, particularly in light of (i) the established role of the microbiota-mucosal immune axis in antitumor immune responses and (ii) the association of tumor progression and poor prognosis in ESA-treated patients (9, 24).

## Data Availability

Data sequencing have been deposite on NCBI under the following BioProject ID: PRJNA1208412.

## Supplementary material

Supplementary Table 1 Complete blood count analysis from mice treated 14, 21 and 28 days with EPO. Mann-Whitney tests. *p < 0.05; **p < 0.01; ***p < 0.001, ****p < 0.0001.

Supplementary Figure 1 Gating strategy for different immune cell populations in the digestive mucosa. **(A)** Gating strategy for intraepithelial lymphocyte populations (IEL). **(B)** Gating strategy for lamina propria lymphocytes (LPL) populations. **(C)** Gating strategy for lamina propria antigen-presenting cells (APC). **(D)** Gating strategy for Peyer’s patches and lamina propria IgA-positive plasma cells.

Supplementary Figure 2 Gating strategy for intracellular IL-10 and IL-17A staining.

Supplementary Figure 3 Macroscopical changes induced by PBS and EPO treatment. Mice were sacrificed on days 14, 21 or 28, and tissue samples were collected. Mice bodyweight, Small intestine and Colon length and Cecum weight/mice bodyweight ratio were evaluated and compared. Each symbol corresponds to an individual mouse (*n* ≥ 10). For each parameter studied, on average four independent experiments were carried out, each involving 3 to 5 mice per group. The data are expressed as the mean ± SD. Mann-Whitney tests. *p < 0.05; **p < 0.01; ***p < 0.001, ****p < 0.0001.

Supplementary Figure 4 Phylum-level taxonomic analysis of fecal microbiota in PBS- and EPO-treated mice. Relative abundances of *Firmicutes*, *Bacteroidota*, *Actinobacteriota*, *Deferribacterota* and *Desulfobacterota* and other phyla in PBS- and EPO-treated mice. Each symbol corresponds to an individual mouse (*n* ≥ 8). (Kruskal-Wallis). Each symbol corresponds to an individual mouse. False discovery rate (FDR)-corrected *P*-values. FDR < 0.05 was considered significant for all tests *p < 0.05; **p < 0.01; ***p < 0.001, ****p < 0.0001.

Supplementary Figure 5 Phylum- and family-level taxonomical analyses of the fecal microbiota in PBS- and EPO-treated mice. Each symbol corresponds to an individual mouse. The significance of correlations between microbiome composition in fecal samples and the hematocrit was established using Spearman’s test (p < 0.05*) (*n* > 20).

Supplementary Figure 6 Frequencies of intraepithelial T lymphocytes (IELs). **(A)** Frequencies of ileal and colonic CD4, CD8, CD4^+^CD8^+^ (DP) and CD4^-^CD8^-^ (DN) Tαβ IELs among CD3-positive cells from PBS- and EPO-treated mice, after 14, 21 and 28 days of treatment. **(B)** Frequency of ileal and colonic CD8^+^ and CD4^-^CD8^-^ (DN) Tγδ IELs among CD3-positive cells from PBS- and EPO-treated mice, after 14, 21 and 28 days of treatment. For each parameter studied, on average four independent experiments were carried out, each involving 3 to 5 mice per group. Each symbol corresponds to an individual mouse. The data are expressed as the mean ± SD. Mann-Whitney tests. *p < 0.05; **p < 0.01; ***p < 0.001, ****p < 0.0001.

Supplementary Figure 7 Frequencies of LPLs. **(A)** Frequency of ileal and colonic CD4, CD8, CD4^+^CD8^+^ (DP) and CD4^-^CD8^-^ (DN) LPLs among CD3-positive cells from PBS- and EPO-treated mice, after 14, 21 and 28 days of treatment (*n* ≥ 8). Ileal **(B)** and colonic **(C)** frequencies of CD4^+^RORγ^+^, CD4^+^Foxp3^+^ and CD4^+^Foxp3^+^RORγ^+^, among CD3-positive cells in PBS- and EPO-treated mice, after 14, 21 and 28 days of treatment (*n* = 10 at day 28). Each symbol corresponds to an individual mouse. The data are expressed as the mean ± SD. Mann-Whitney tests. *p < 0.05; **p < 0.01; ***p < 0.001, ****p < 0.0001.

Supplementary Figure 8 Frequencies of follicular T helper lymphocytes from Peyer’s patches. **(A)** Gating strategy for CD4 (TfhCD4^+^) and CD8 (TfhCD8^+^) follicular T helper lymphocytes from Peyer’s patches. **(B)** Frequencies of TfhCD4^+^ (CD3^+^CD4^+^CD44^+^CXCR5^+^PD1^+^) and TfhCD8^+^ (CD3^+^CD4^-^CD44^+^CXCR5^+^PD1^+^) cells from Peyer’s patches among CD3-positive cells (*n* = 10). Each symbol corresponds to an individual mouse. The data are expressed as the mean ± SD. Mann-Whitney tests. *p < 0.05; **p < 0.01; ***p < 0.001, ****p < 0.0001.

Supplementary Figure 9 Frequencies LP mucosal antigen-presenting cells: **(A)** Colonic and ileal APCs frequencies among lineage-negative cells. Ileal **(B)** and colonic **(C)** subpopulations of APCs subpopulations among CD45^+^MHC-II^+^ cells from PBS- and EPO-treated mice, after 14, 21 and 28 days of treatment. Each symbol corresponds to an individual mouse. The data are expressed as the mean ± SD. Mann-Whitney tests. *p < 0.05; **p < 0.01; ***p < 0.001, ****p < 0.0001 (*n* ≥ 8).
