# Supplementary material for: Erythropoietin supplementation induces dysbiosis of the gut microbiota and impacts mucosal immunity in a non-diseased mouse model
Source: Front Immunol. 2025 Jan 23;15:1465410. doi: 10.3389/fimmu.2024.1465410 (PMC11798978; doi:10.3389/fimmu.2024.1465410)

Supplementary Table 1

	day 14			day 21			day 28		
	PBS	EPO	P value	PBS	EPO	P value	PBS	EPO	P value
WBC	7,1 (4 ; 10,1)	9,5 (6,2 ; 13,2)	0,17	7,1 (4,1 ; 12)	10,6 (3,7 ; 21,9)	0,13	9,1 (5 ; 11,1)	8,5 (1,9 ; 12,2)	0,83
RBC	8,4 (6,7 ; 9,2)	11,4 (10,8 ; 12,7)	0,0001	8,3 (7 ; 9,5)	11,2 (10,1 ; 12,6)	0,0001	8,3 (7 ; 9,2)	11,7 (10,4 ; 13,7)	0,0001
MCV	44,8 (42,3 ; 50,2)	49 (42,7 ; 54,7)	0,04	44,7 (43 ; 46,2)	48 (46,3 ; 50,4)	0,0001	43 (41,7 ; 44,8)	47,7 (44,4 ; 50,3)	0,0001
Hct	37,4 (29 ; 42,2)	56 (46,5 ; 60,1)	0,0001	37,2 (31 ; 43)	53,6 (46,6 ; 59,4)	0,0001	35,6 (29,4 ; 40)	55 (47 ; 70)	0,0001
Hgb	13,3 (10,4 ; 14,4)	19,4 (16,8 ; 21,4)	0,0001	13 (10,7 ; 14,5)	19 (16,5 ; 20,7)	0,0001	12,8 (11,1 ; 14,7)	19,9 (17,6 ; 22,2)	0,0001
PLT	905 (50,3 ; 1305)	788 (329 ; 1076)	0,16	1034 (770 ; 71339)	897 (586 ; 1361)	0,036	996 (352 ; 1383)	848 (665 ; 1287)	0,18
Lym%	82,6 (77 ; 89,3)	73,1 (66,5 ; 81,3)	0,0001	80,3 (71,4 ; 88,2)	73,7 (66,1 ; 81,5)	0,008	82 (74 ; 85,8)	68,5 (21,4 ; 82,1)	0,0006
Mon%	3,6 (2 ; 4,5)	6,6 (2,3 ; 12,6)	0,04	4,2 (2,3 ; 5,9)	7,8 (5,6 ; 10,7)	0,0001	4,4 (3,4 ; 5,5)	7,3 (1,7 ; 12,7)	0,001
N/Gr%	9,1 (7,3 ; 14,5)	15 (10,2 ; 22,1)	0,012	11,1 (5,3 ; 17,7)	11,9 (7,3 ; 16,4)	0,59	9 (5,2 ; 14)	16 (5,6 ; 18,8)	0,52
Eos%	1,7 (0 ; 8,5)	0,5 (0 ; 1,6)	0,56	1,2 (0 ; 11)	0,9 (0,1 ; 2,6)	0,48	1,5 (0 ; 7,3)	2,6 (0,3 ; 5,9)	0,1
Bas%	1,7 (0 ; 2,5)	2,2 (0,5 ; 3,3)	0,11	2,1 (1,4 ; 2,8)	2,5 (1,8 ; 3)	0,02	1,9 (1,4 ; 2,4)	2,3 (0,9 ; 2,8)	0,02
Unid%	1,2 (0,7 ; 1,8)	2,5 (1,2 ; 4,1)	0,001	1,5 (0,5 ; 2,9)	3,2 (2 ; 6,5)	0,0003	1,3 (0,9 ; 1,7)	2,9 (0 ; 10,9)	0,005

Supplementary Figure 1

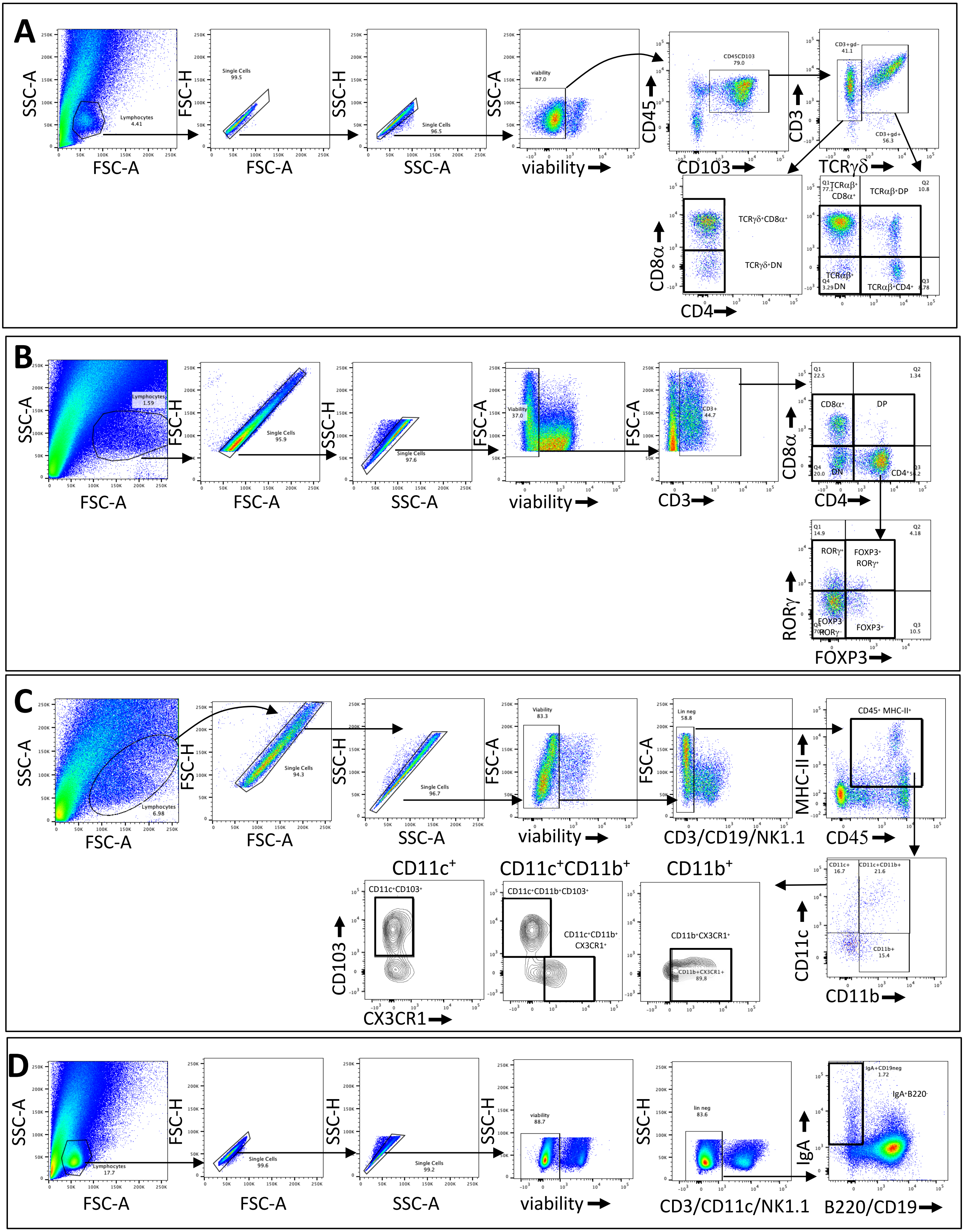

Supplementary Figure 2

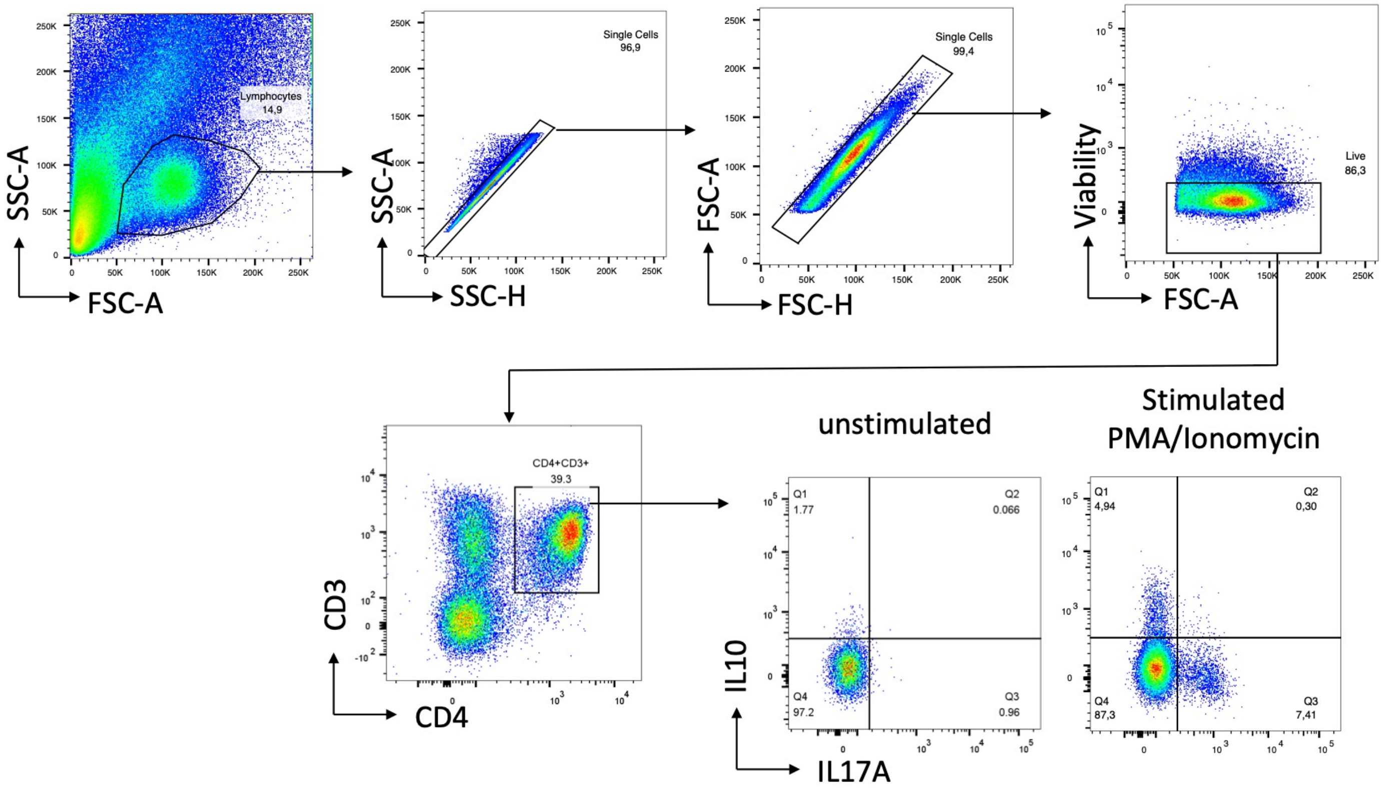

Supplementary Figure 3

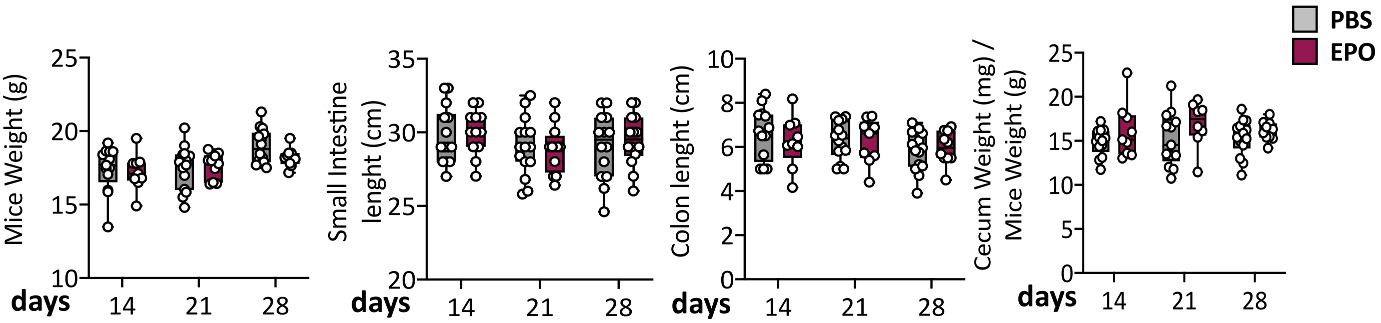

Supplementary Figure 4

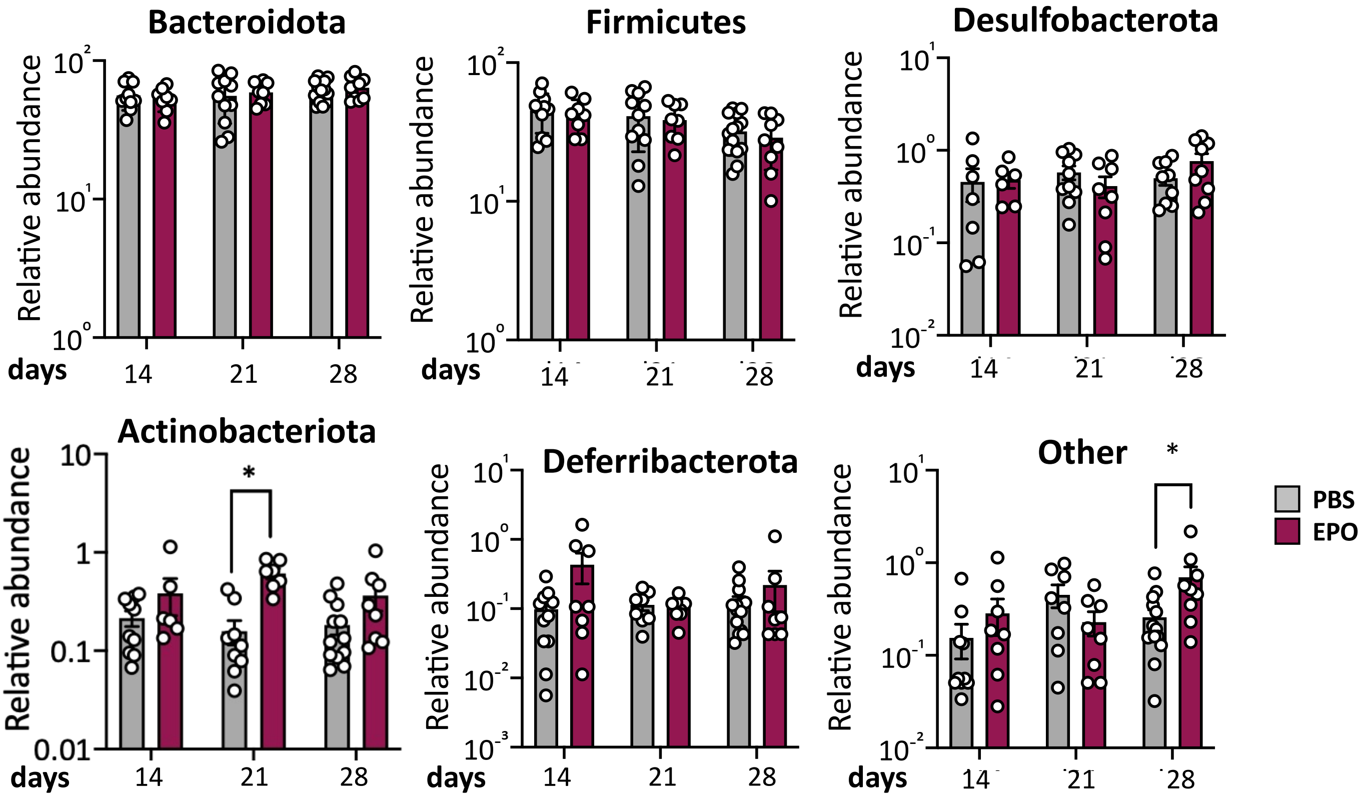

Supplementary Figure 5

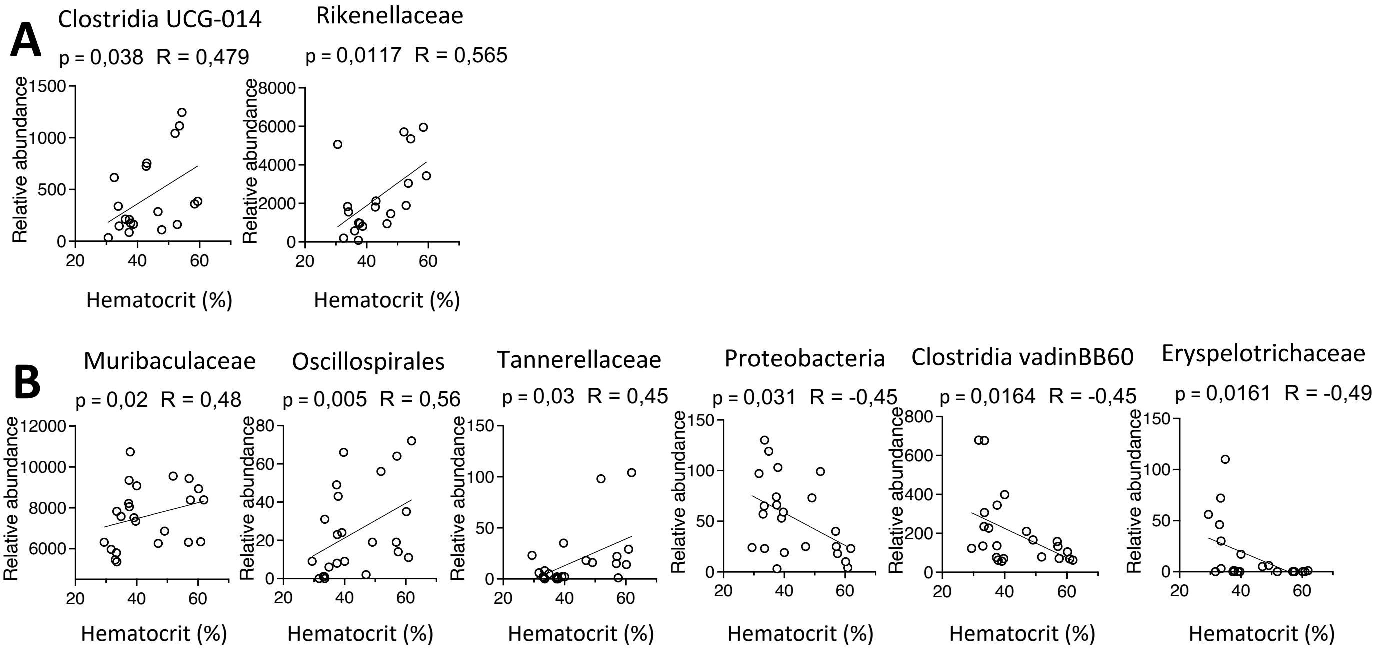

Supplementary Figure 6

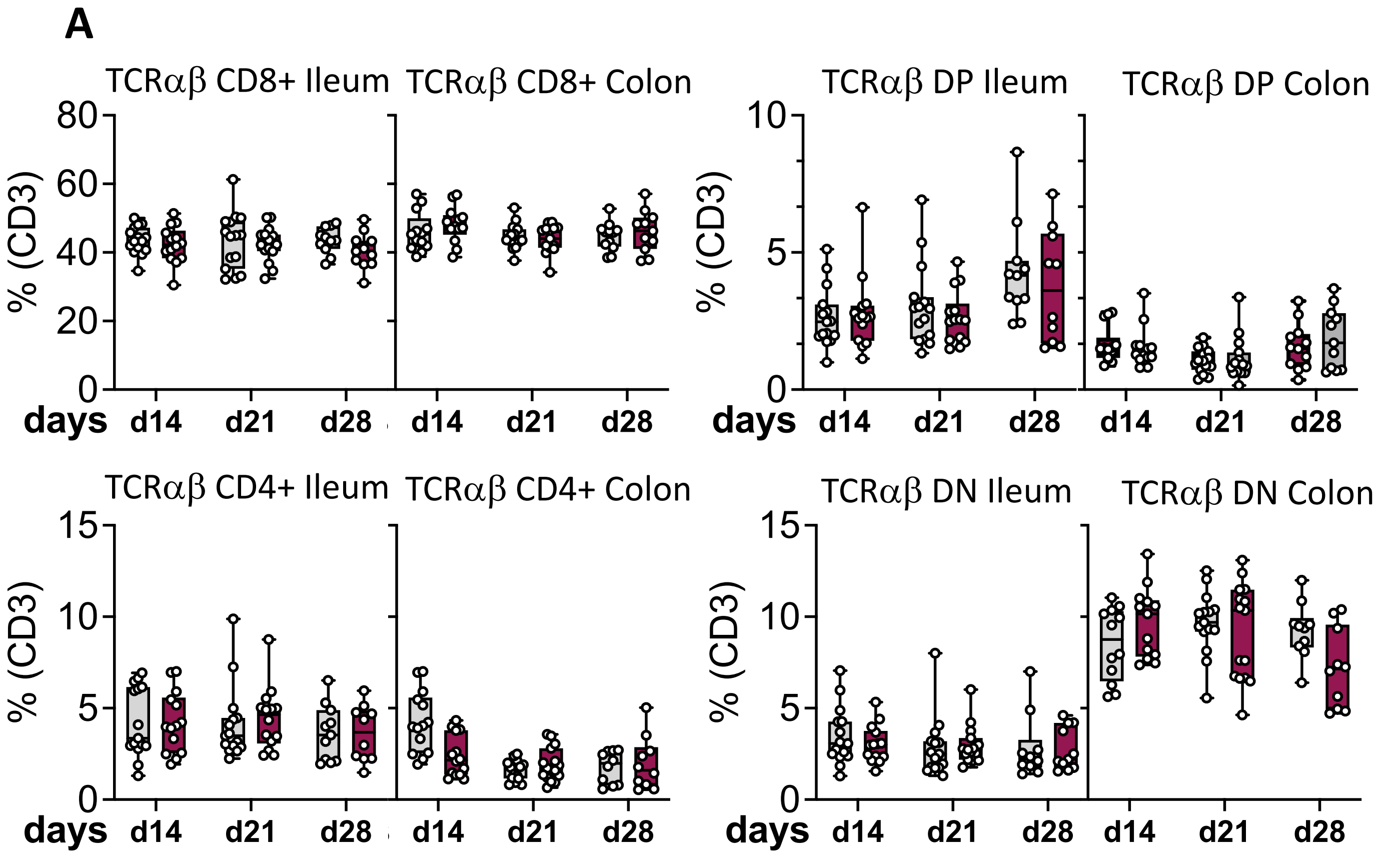

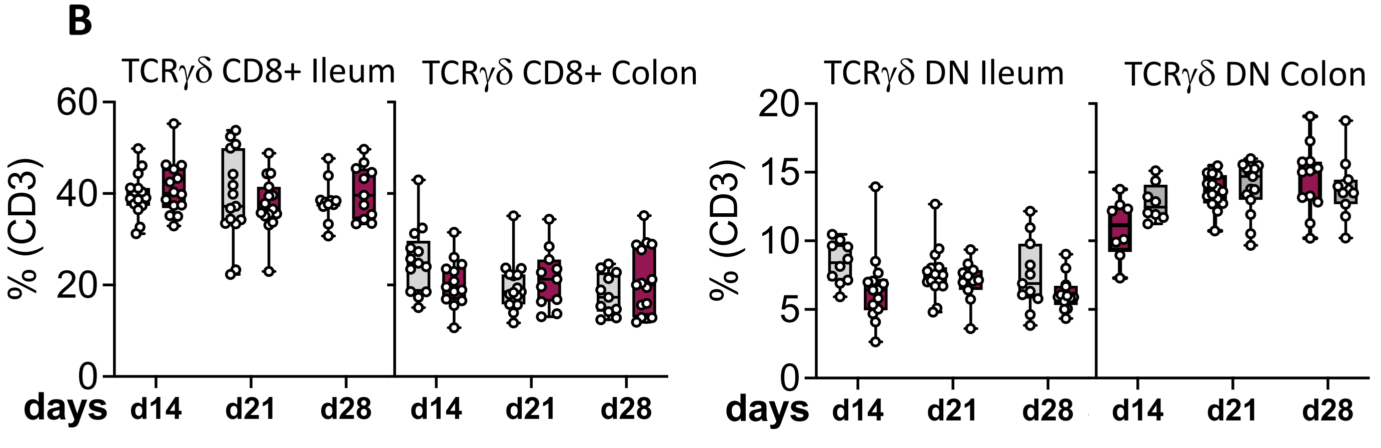

Supplementary Figure 7

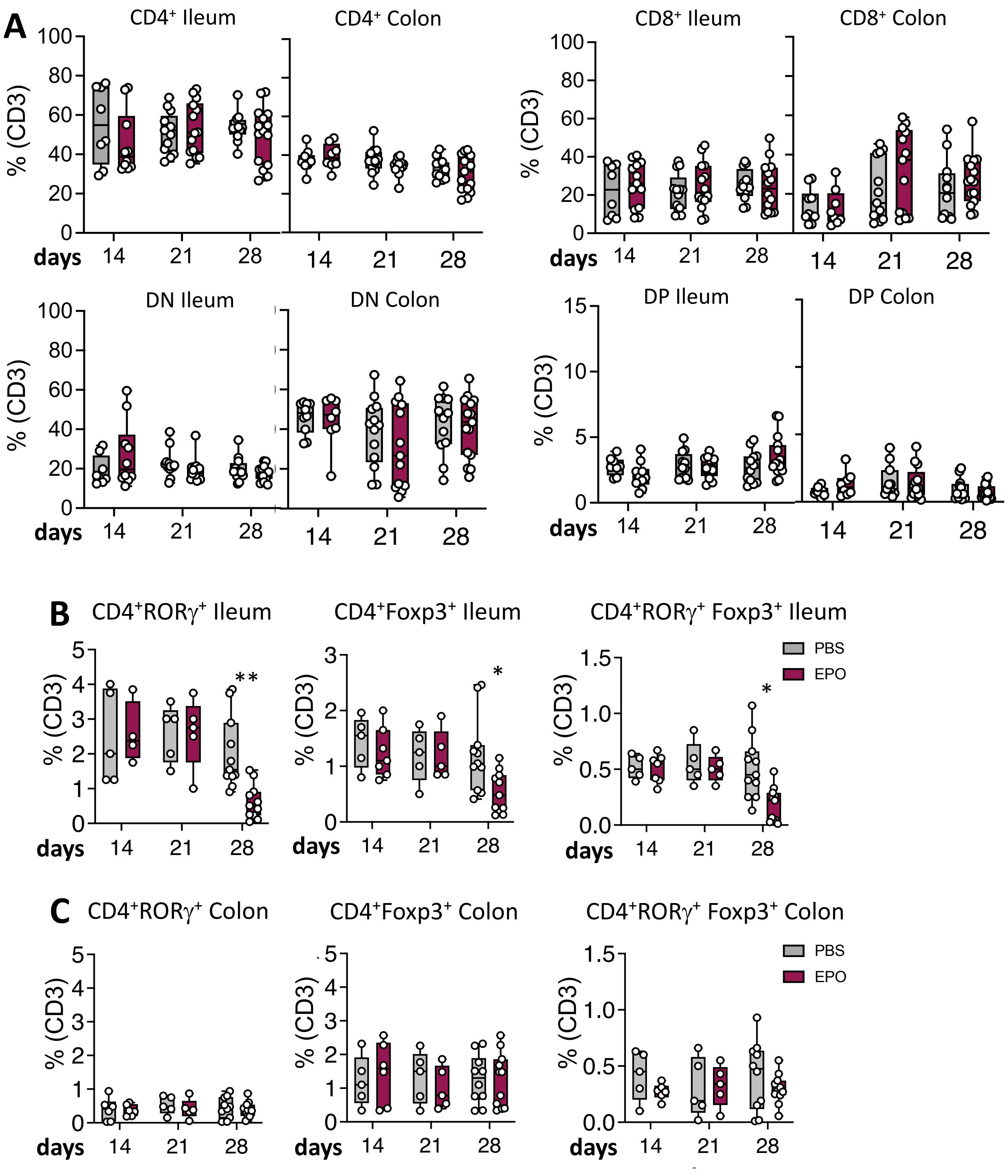

Supplementary Figure 8

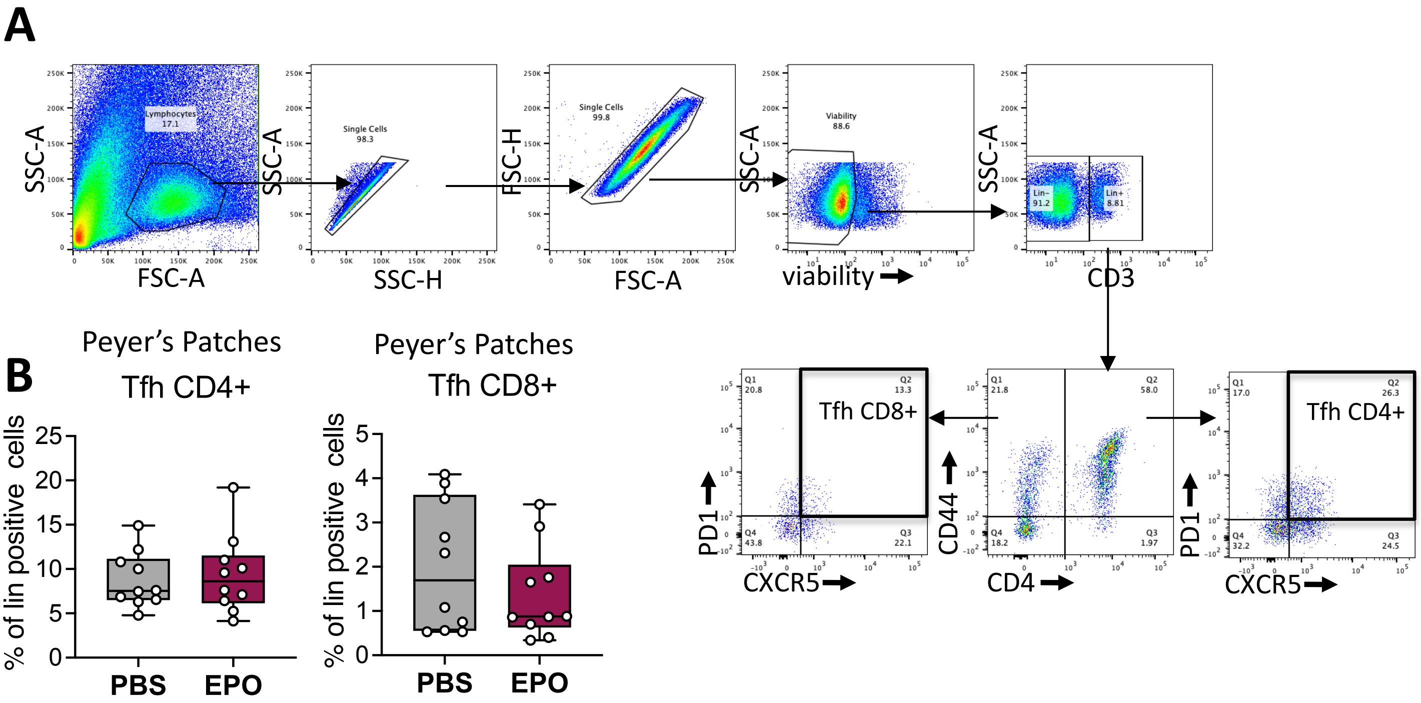

Supplementary Figure 9

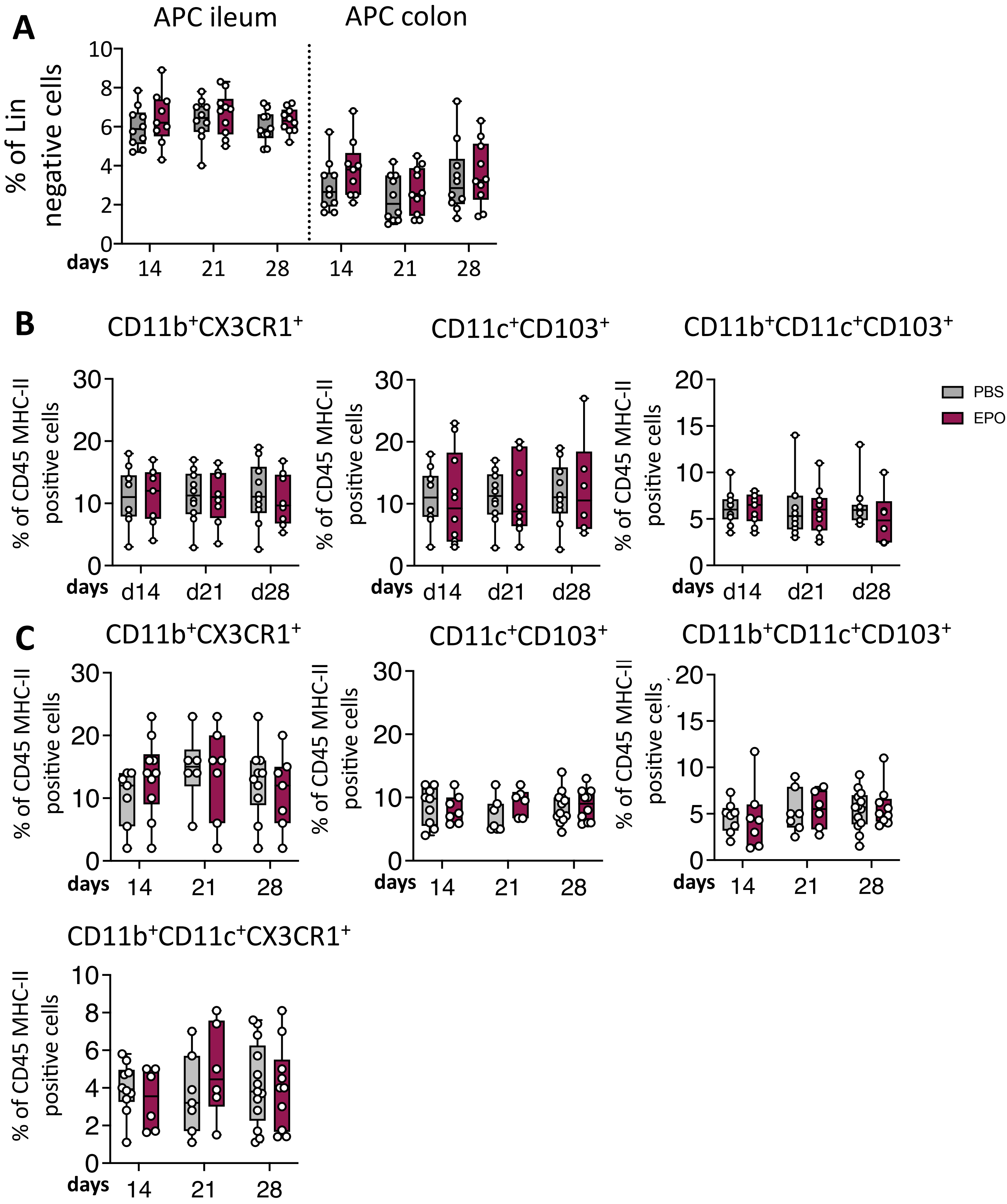

Supplement: Supplementary Table 1 — Complete blood count analysis from mice treated 14, 21 and 28 days with EPO. Mann-Whitney tests. *p < 0.05; **p < 0.01; ***p < 0.001, ****p < 0.0001. [file DataSheet1.pdf]
