# Supplementary material for: Erythropoietin supplementation induces dysbiosis of the gut microbiota and impacts mucosal immunity in a non-diseased mouse model
Source: Front Immunol. 2025 Jan 23;15:1465410. doi: 10.3389/fimmu.2024.1465410 (PMC11798978; doi:10.3389/fimmu.2024.1465410)
Supplement: Supplementary file 2 [file DataSheet2.pdf]

Supplementary Material

Antibody Name	Target	Fluorochrom	Clone	Reference	Supplier	Staining Mix
Anti-Mouse gamma delta TCR FITC	gd TCR	FITC	GL3	11-5711-	ebioscience	IEL
Anti-Human/Mouse CD45R (B220) PerCP-Cyanine5.5	CD45R/B220/Ly-5	PercP-Cy5.5	RA3-6B2	45-0452	ebioscience	Plasma cells
Anti-Mouse CXCR5-PERCP efluor 710	CD185/ CXCR5	PerCP-Cy5.5	L138D4	145508	biolegend	Tfh
Anti-Mouse MHC II PerCP-Cyanine5.5	CMH II (I-A/I-E)	PercP-Cy5.5	M5/114,15,2	107626	biolegend	APC LPL
Anti-Mouse CD11c PE-Dazzle 594	CD11c	PE-Dazzle 594	N418	BLE117347	biolegend	APC LPL
Anti mouse CD103 PE/Dazzle 594	CD103	PE/Dazzle 594	2E7	BLE121429	biolegend	IEL
Anti-Mouse CD45 PE	CD45	PE	30F11	130-102-596	miltenyi	IEL/APC LPL
Anti-Mouse IgA PE	IgA	PE	mA-6E1	12-4204	ebioscience	Plasma cells
Anti-Mouse CD44-PE-Cyanine 5	CD44	PE-Cy5	IM7	103010	biolegend	Plasma cells
Anti-Mouse CD19 PercP-Cy5.5	CD19	PercP-Cy5.5	1D3	25-0193	ebioscience	Plasma cells
Anti-mouse CX3CR1	CX3CR1	PE-Cy7	SA011F11	149016	biolegend	APC LPL
Anti-Mouse CD103-APC	CD103	APC	2E7	BLE121414	biolegend	APC LPL
Anti-Mouse/Rat Foxp3 APC	FoxP3	APC	FJK-16s	17-5773	ebioscience	T cells LPL
Anti-Mouse CD8a AF700	CD8a	Alexa Fluor 700	53-6.7	BLE100730	biolegend	IEL / T cells LPL / Tfh
Anti-Mouse CD11b AF700	CD11b	Alexa Fluor 700	M1/70	BLE101222	biolegend	APC LPL
Anti-Mouse CD4 BV 421	CD4	BV 421	GK1.5	100438	biolegend	IEL / T cells LPL
Fixable Viability dye eFluor 506		eFluor506		65-0866-18	ebioscience	All
Anti mouse RORgt	RORgt	BV650	Q31-378	564722	biolegend	T cells LPL
Anti-Mouse CD3 Brilliant Violet 711	CD3	BV 711	17A2	BLE 100241	biolegend	All
Anti mouse CD11c BV 711	CD11c	BV711	N418	117349	biolegend	Plasma cells
Anti-Mouse CD19 Brilliant Violet 711	CD19	BV 711	6D5	BLE115555	biolegend	APC LPL
Anti mouse NK1.1 BV 711	NK1.1	BV711	PK136	108745	biolegend	APC LPL
Anti-Mouse PD1 APC e-Fluor 780	PD1	APC e-Fluor 780	J43	47-9985	ebioscience	Tfh
Anti-Mouse IL-10 APC-Cy7	IL-10	APC-Cy7	JESS-116E3	BLE503036	biolegend	T cells LPL (activation)
Anti-Mouse IL-17A PE-Cyanine7	IL-17A	PE-Cyanine7	17B7	25-7177	ebioscience	T cells LPL (activation)
